# Embolization of a Superior Thyroid Artery Hemorrhage after Fine-Needle Aspiration Biopsy of a Thyroid Nodule

**DOI:** 10.1155/2020/3727696

**Published:** 2020-04-06

**Authors:** Christopher Gates, Maxwell Newby, William Stokes, SoHyun Boo, Michele Carr

**Affiliations:** ^1^Department of Otolaryngology, Head and Neck Surgery, West Virginia University School of Medicine, Robert C. Byrd Health Sciences Center, Morgantown, West Virginia, USA; ^2^Department of Interventional Neuroradiology, West Virginia University School of Medicine, Robert C. Byrd Health Sciences Center, Morgantown, West Virginia, USA

## Abstract

Fine-needle aspiration biopsy (FNAB) is a procedure completed thousands of times daily across the world as an efficacious and safe way to evaluate thyroid nodules. Complications of an FNAB typically range from patient intolerance to small intrathyroidal hematomas. In rare situations, an FNA may result in significant bleeding leading to airway compromise or significant blood loss. In this case report, a patient underwent an FNAB and developed an arterial bleed leading to an intrathyroidal hematoma and airway compromise requiring intubation. This case report is unique in that it identifies the source of bleeding, exemplifies the complications of a large intrathyroidal hematoma, and describes subsequent treatment of both the arterial bleed and the hematoma.

## 1. Introduction

Fine-needle aspiration biopsy (FNAB) is a diagnostic modality that has a track record of proven safety, economy, and efficacy for evaluation of thyroid nodules. The procedure is commonly performed in outpatient settings with local anesthetic and a 22 to 27 gauge needle. FNAB has reduced the number of thyroidectomies showing no malignant pathology [1, 2]. Common complications include patient discomfort and microhematomas within the thyroid gland [3, 4]. Hematomas are likely a result of the highly vascular nature of the thyroid, arteriovenous shunts, and weakened venous vessels. If detected, hematomas are often minor and require only short-term compression [3].

To date, there have been a handful of cases of massive thyroid hematoma after FNAB reported in the literature [5–11]. Few have resulted in airway compromise that required urgent intubation [7, 8, 11]. Our group reports a case of significant injury to the superior thyroid artery where the patient was intubated for airway protection. This case is unique in that it identifies the source of the bleeding, which is an example of complications of a large intrathyroidal hematoma, and describes subsequent treatment of both the arterial bleed and the hematoma.

## 2. Case Details

The patient is a 31-year-old male with a history of hypertension, hypothyroidism, and hyperlipidemia who initially presented to his primary care provider with fatigue and lethargy. He had no past surgical history and his family medical history was noncontributory. He was found to have severe hypothyroidism with a thyroid stimulating hormone level of greater than 100. He presented to the Emergency Department (ED) at an outside hospital for concerns of ongoing leg swelling, fatigue, and lethargy in conjunction with hypothyroidism. Upon admission to the ED, he was found to have a deep vein thrombosis (DVT) in the popliteal vein. He was then started on enoxaparin sodium 100 milligrams twice a day and was scheduled for a thyroid ultrasound. The thyroid ultrasound displayed multiple bilateral hypoechoic thyroid nodules with the largest 3.5 cm in diameter. He underwent FNAB of the dominant left and right nodules with 5 passes each, using a 22 gauge needle under ultrasound guidance.

**Figure 1:**
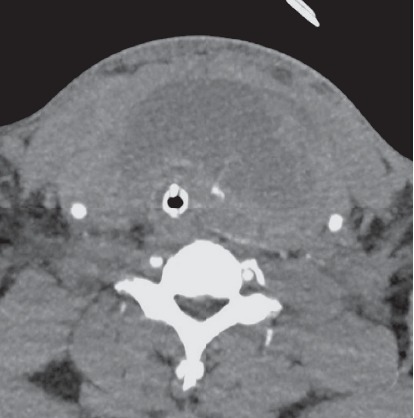
Axial CTA scan demonstrating thyroid hematoma and collapsed trachea with arterial blush concerning for active hemorrhage in the thyroid parenchyma.

**Figure 2:**
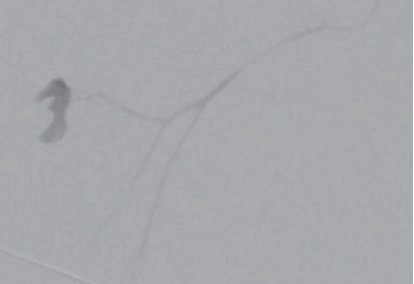
Angiography demonstrating blush in the left superior thyroid artery, raising concerns for an active hemorrhage into the thyroid.

**Figure 3:**
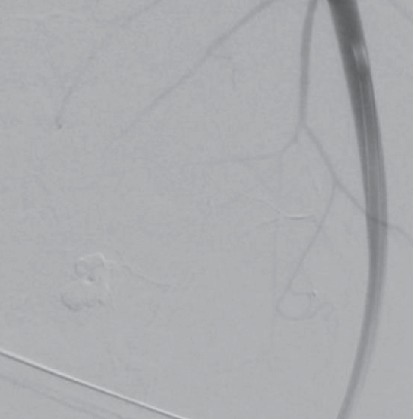
Angiography of the left thyroid vessels following embolization. The blush is no longer present, indicating success of the embolization.

The procedure was reported to have gone well; however, several hours later he developed anterior neck swelling and difficulty breathing. An ultrasound at that time confirmed an intrathyroidal hematoma with concern for arterial disruption. The patient was fiberoptically intubated at the outside hospital for airway protection and transferred to a tertiary care center. Upon admission, computed tomographic angiography of the neck displayed a large intrathyroidal hematoma with an arterial blush concerning for active hemorrhage (Figure 1). Enoxaparin sodium was held at the time of his admission to the tertiary care center. Interventional radiology was consulted for possible embolization of the hemorrhaging vessel. The patient was found to have active bleeding from his left superior thyroid artery (Figure 2), which was successfully embolized using n-butyl cyanoacrylate particles (Figure 3). The patient was assessed for an endotracheal cuff leak the next day for possible extubation. Unfortunately, the patient did not have a leak and the hematoma was not noted to be reduced in size. He was then taken to the operating room the next day for evacuation of the intrathyroidal hematoma to expedite extubation.

The patient was then taken to the operating room the next day for evacuation of the intrathyroidal hematoma. A 4 centimeter transcervical incision was made over the lower anterior neck and exploration identified an organized intrathyroidal hematoma with no active bleeding. The hematoma was noted to be superficially involving the thyroid but it appeared viable and thyroidectomy was not deemed necessary. The neck was then copiously irrigated.

A Jackson-Pratt drain was placed in the wound bed. Enoxaparin sodium was restarted on postoperative day one after his neck evacuation and the patient was extubated. The patient had no further shortness of breath. He was subsequently discharged on postoperative day two after an uneventful hospital stay. His Jackson-Pratt drain was removed in clinic on postoperative day five. His surgical incision appeared to be healing well and neck soft without reaccumulation of fluid.

## 3. Review of the Literature

The most common adverse events associated with fine-needle aspirations stem from patient intolerance or minor hematomas [3]. Overall, it is a well-tolerated procedure that is successfully completed in outpatient settings. However, few cases have been reported where a hematoma required intubation.

Theoretically, a 22-gauge needle will have an increased hemorrhage rate when compared to 25 or 27 gauge needles. Increased needle diameter and multiple passes may have contributed to hematoma formation in this case. However, due to the rarity of its occurrence, a literature search found no association of airway compromise secondary to hematoma formation and needle gauge. One other group did report a thyroid hematoma with a 22-gauge needle that required surgery but not intubation [6]. In addition, a systematic review by Moss et al. found a decreased rate of nondiagnostic samples and patient discomfort when small 25 and 27 gauge needles were used with capillary action rather than negative pressure to obtain tissue biopsy [12]. Even with an accidental arterial injury a smaller gauge needle may have allowed for spontaneous closure of the vessel defect and decreased the overall hemorrhage. This patient was also taking 100 milligrams of enoxaparin sodium twice a day as treatment of his DVT at the time the FNAB was performed. The literature has shown that anticoagulation with FNAB is a topic of debate, but does appear to be safe in the available retrospective data [13]. Khadra et al. demonstrated no increased rate or severity of bleeding associated with the FNAB on therapeutic anticoagulation in their series of almost 1600 patients [13]. In the case of this patient, the bleeding was found to be arterial in nature, and although the enoxaparin sodium therapy may have affected the time to stopping this hemorrhage it likely was not the underlying cause.

A comparison of needle gauge and total passes was completed using data from previously reported cases to establish a possible trend. Despite more than a dozen reported hematomas post-FNAB that resulted in airway compromise, few cases were found with data regarding needle gauge, number of passes, and requirement of intubation. Those that are available can be found in Table 1 and show needles with gauges between 21 and 25 were used with a total number of passes ranging from 2 to 10, and half of the patients required intubation. Of particular interest is that all of these patients experienced airway discomfort, yet almost half of them were successfully managed without endotracheal intubation. Even if intubation was not required, most were managed surgically through thyroidectomy or ligation of a vessel. These studies demonstrate that expanding hematomas that require urgent medical attention are possible with a low number of passes and most needle gauges.

Finally, this case stresses the importance of immediate assessment of the airway and possibly prophylactic intubation in any patient who develops bleeding in the head and neck region. This patient had a rapidly expanding hematoma of his neck that resulted in airway compromise. The nature of the neck swelling made intubation difficult and the need for fiberoptic assistance. Additionally, the large neck swelling from the hematoma would have blocked surgical access to the trachea and cricothyroid membrane making establishing a surgical airway difficult if not impossible. Roh et al. describe a similar situation in their report of an intrathyroidal hematoma resulting in airway compromise and requiring elective intubation, and deaths have been reported with thyroid hematomas after core needle biopsies due to airway compromise [8, 14]. Despite this patient's rapid deterioration and requirement of aggressive management, FNAB is still a reliable and safe procedure. However, physicians recommending and performing this procedure should remain cognizant of the associated risks of FNAB of thyroid nodules. Use of smaller gauge needles and consideration to discontinue anticoagulation therapy in low risk patients prior to FNABs may have prevented this complication. Additionally, the patients that report sudden onset dysphagia or difficulty breathing after FNAB should be promptly evaluated, and preference should be given to immediate control of the airway prior to further intervention.

## 4. Conclusions

Though FNAB of thyroid nodules is typically a safe efficacious procedure, it is not without risk. This case highlights the rare but serious adverse event of arterial injury with subsequent hematoma formation and airway compromise. Our case is unique in that the causal artery was identified and embolized, followed by surgical management of the hematoma. It presents a rare occurrence that has the possibility of permanent harm or death. Prior to discharge, the patients must be educated regarding when to seek help, and physicians should maintain a low threshold for protecting airways if complications arise.

## Figures and Tables

**Table 1:** Characteristics of procedures that resulted in thyroid hematoma post-FNAB, including needle gauge, number of nodules, passes per nodule, and intubation status.

Study	Needle gauge	Number of nodules	Number of passes per nodule	Taking anticoagulant	Clotting disorder	Intubation required	Management
Noordzij and Goto [7]	25	1	6	No	No	No	Surgical evacuation and cautery
Katagiri et. al. [6]	22	1	2	No	No	No	Surgical evacuation and ligation of superior thyroid artery
Park and Yoon [11]	24	1	Unknown	Unknown	Unknown	Yes	Embolization of superior thyroid artery
Roh [8]	Unknown	Bilateral	Unknown	No	No	Yes	Surgical exploration with thyroidectomy
Lee [10]	Unknown	Unknown	Unknown	No	No	No	Neck compression and observation
Hor and Lahiri [5]	25	2	3	325 mg aspirin	No	Yes	Surgical exploration with thyroid isthmusectomy
Veverková et al. [9]	21	1	3	Nadroparin calcium	Unknown	Yes	Surgical evacuation, ligation of superior thyroid artery, and right hemithyroidectomy
Current case	22	2	5	Enoxaparin sodium	No	Yes	Embolization followed by surgical evacuation of hematoma
